# The prognosis and metabolite changes of NSCLC patients receiving first‐line immunotherapy combined chemotherapy in different M1c categories according to 9th edition of TNM classification

**DOI:** 10.1002/cam4.70223

**Published:** 2024-09-11

**Authors:** Liang Zheng, Fang Hu, Wei Nie, Jun Lu, Bo Zhang, Jianlin Xu, Shuyuan Wang, Ying Li, Xiaoxuan Zheng, Wei Zhang, Yinchen Shen, Runbo Zhong, Tianqing Chu, Baohui Han, Hua Zhong, Xueyan Zhang

**Affiliations:** ^1^ Department of Respiratory and Critical Care Medicine, Shanghai Chest Hospital Shanghai Jiao Tong University School of Medicine Shanghai China; ^2^ Department of Thoracic Medical Oncology The Cancer Hospital of the University of Chinese Academy of Sciences (Zhejiang Cancer Hospital) Zhejiang China; ^3^ Hangzhou Institute of Medicine (HlM) Chinese Academy of Sciences Zhejiang China

**Keywords:** 9th edition TNM classification, immune‐checkpoint inhibitors, non‐small cell lung cancer, prognosis, untargeted metabolomics

## Abstract

**Background:**

The 9th edition of the TNM Classification for lung cancer delineates M1c into two subcategories: M1c1 (Multiple extrathoracic lesions within a single organ system) and M1c2 (Multiple extrathoracic lesions involving multiple organ systems). Existing research indicates that patients with lung cancer in stage M1c1 exhibit superior overall survival compared to those in stage M1c2. The primary frontline therapy for patients with advanced non‐small cell lung cancer (NSCLC), lacking driver gene mutations, involves the use of immune checkpoint inhibitors (ICIs) combined with chemotherapy. Nevertheless, a dearth of evidence exists regarding potential survival disparities between NSCLC patients with M1c1 and M1c2 undergoing first‐line immune‐chemotherapy, and reliable biomarkers for predicting treatment outcomes are elusive. Serum metabolic profiles may elucidate distinct prognostic mechanisms, necessitating the identification of divergent metabolites in M1c1 and M1c2 undergoing combination therapy. This study seeks to scrutinize survival discrepancies between various metastatic patterns (M1c1 and M1c2) and pinpoint metabolites associated with treatment outcomes in NSCLC patients undergoing first‐line ICIs combined with chemotherapy.

**Method:**

In this study, 33 NSCLC patients lacking driver gene mutations diagnosed with M1c1, and 22 similarly diagnosed with M1c2 according to the 9th edition of TNM Classification, were enrolled. These patients received first‐line PD‐1 inhibitor plus chemotherapy. The relationship between metastatic patterns and progression‐free survival (PFS) in patients undergoing combination therapy was analyzed using univariate and multivariate Cox regression models. Serum samples were obtained from all patients before treatment initiation for untargeted metabolomics analysis, aiming to identify differential metabolites.

**Results:**

In the univariate analysis of PFS, NSCLC patients in M1c1 receiving first‐line PD‐1 inhibitor plus chemotherapy exhibited an extended PFS (HR = 0.49, 95% CI, 0.27–0.88, *p* = 0.017). In multivariate PFS analyses, these M1c1 patients receiving first‐line PD‐1 inhibitor plus chemotherapy also demonstrated prolonged PFS (HR = 0.45, 95% CI, 0.22–0.92, *p* = 0.028). The serum metabolic profiles of M1c1 and M1c2 undergoing first‐line PD‐1 inhibitors plus chemotherapy displayed notable distinctions. In comparison to M1c1 patients, M1c2 patients exhibited alterations in various pathways pretreatment, including platelet activation, linoleic acid metabolism, and the VEGF signaling pathway. Diminished levels of lipid‐associated metabolites (diacylglycerol, sphingomyelin) were correlated with adverse outcomes.

**Conclusion:**

NSCLC patients in M1c1, devoid of driver gene mutations, receiving first‐line PD‐1 inhibitors combined with chemotherapy, experienced superior outcomes compared to M1c2 patients. Moreover, metabolomic profiles strongly correlated with the prognosis of these patients, and M1c2 patients with unfavorable outcomes manifested distinct changes in metabolic pathways before treatment. These changes predominantly involved alterations in lipid metabolism, such as decreased diacylglycerol and sphingomyelin, which may impact tumor migration and invasion.

## 1 INTRODUCTION

Lung cancer represents a significant health challenge, with high incidence and mortality rates in China and globally. Non‐small cell lung cancer (NSCLC) constitutes approximately 85% of all lung cancer cases.^1^, ^2^ TNM staging, based on tumor size (T), lymph node metastasis (N), and distant organ metastasis (M), serves as a standard method to assess lung cancer severity, predict patient prognosis, and guide treatment decisions.^3^ The 9th edition of TNM Classification for lung cancer, introduced at the 2023 World Conference on Lung Cancer, further refines M1c into two subcategories: M1c1 (multiple extrathoracic metastases within a single organ system) and M1c2 (multiple extrathoracic metastases involving multiple organ systems). Evidence indicates distinct survival curves for M1c1 and M1c2.^4^

Programmed cell death 1/programmed cell death ligand‐1 (PD‐1/PD‐L1) inhibitor combined with chemotherapy has emerged as the primary first‐line treatment for driver gene‐negative NSCLC patients with distant metastasis.^5^, ^6^ However, existing studies have not established divergent prognoses between NSCLC patients with M1c1 and M1c2 receiving first‐line immunotherapy combined with chemotherapy. Biomarkers for predicting outcomes in immunotherapy combined chemotherapy remain to be elucidated. While PD‐L1 expression serves as a pivotal biomarker for immunotherapy efficacy prediction, patients lacking PD‐L1 expression can still achieve positive outcomes with immunotherapy.^7^ PD‐L1 expression, however, does not perfectly predict immunotherapy efficacy, and factors like temporal and spatial tumor heterogeneity partially account for this variability.^8^, ^9^ Therefore, additional biomarkers are essential for accurate immunotherapy efficacy prediction. Presently, exploring potential blood‐based biomarkers has become a focal point in the lung cancer field,^10^ with these easily accessible and cost‐effective indicators poised to become the next widely used ICIs predictive biomarkers after PD‐L1.

Metabolites, serving as end‐products of biological processes, accurately reflect alterations in cellular function. Various diseases, including lung cancer, are linked to physiological imbalances or the impairment of cellular function arising from shifts in intracellular metabolic states.^11^, ^12^, ^13^ Notably, untargeted metabolomics employs LC–MS, GC–MS, NMR technology for unbiased detection of dynamic changes in all small molecule metabolites (primarily endogenous compounds with a molecular weight under 1000 Da) pre and poststimulation or perturbation in cells, tissues, organs, or organisms. Bioinformatics analysis and pathway analysis of differential metabolites unveil the physiological mechanisms governing these changes. Several studies affirm that untargeted metabolomics analysis of blood samples holds promise for screening novel biomarkers on a large scale for early prediction, diagnosis, and disease classification.^14^, ^15^

Hence, this study aimed to investigate whether NSCLC patients lacking driver gene mutations, classified as M1c1 or M1c2, and treated with first‐line PD‐1/PD‐L1 inhibitors combined with chemotherapy, exhibit divergent prognoses. Additionally, we utilized serum untargeted metabolomics assays to discern disparities in metabolite changes occurring in M1c1 and M1c2 patients before initiating first‐line PD‐1/PD‐L1 inhibitors combined with chemotherapy. The objective is to identify biomarkers capable of predicting patient prognosis.

## 2 METHODS

### 2.1 Patients

Patients with NSCLC lacking driver genes, diagnosed at Shanghai Chest Hospital between January 2019 and December 2021, were retrospectively enrolled. The follow‐up concluded in June 2023, spanning a median duration of 25.6 months. Subsequently, based on the ninth edition of TNM stage, patients were categorized into two groups—M1c1 or M1c2 at the initial diagnosis, comprising 33 with M1c1 and 22 with M1c2. These individuals underwent treatment with first‐line PD‐1/PD‐L1 inhibitors combined with chemotherapy.

The detailed inclusion criteria encompassed: (1) NSCLC diagnosis via histopathology or cytology; (2) M1 stage classification as per the 9th edition TNM criteria, specifically M1c1 or M1c2; (3) age ranging from 18 to 75 years at the time of informed consent, with an ECOG performance status of 0 to 2; (4) presence of at least one measurable lesion according to RECIST 1.1; and (5) receipt of first‐line PD‐1/PD‐L1 inhibitors in conjunction with chemotherapy. Exclusion criteria were defined as: (1) the presence of EGFR/ROS1/ALK mutation; (2) missing data, including failure to complete essential examinations like chest CT and brain MRI; (3) severe underlying diseases or active autoimmune conditions; (4) receipt of hormone and antibiotic therapy within 7 days before serum sample collection; and (5) loss of follow‐up.

The Institutional Review Board of Shanghai Chest Hospital sanctioned this study, with informed consent obtained from all participating patients.

### 2.2 Treatment and data collection

All patients were treated with first‐line PD‐1 or PD‐L1 inhibitors in combination with chemotherapy. In our study, immunotherapeutic agents included pembrolizumab, tislelizumab, sintilimab, atezolizumab, and dovarumab. Among them, the dosage of pembrolizumab, tislelizumab, sintilimab was 200 mg intravenously every 3–4 weeks. The dose of atezolizumab is 1200 mg administered intravenously every 3–4 weeks, while the dose of dovarumab is 1500 mg administered intravenously every 3–4 weeks. The chemotherapy regimen involved platinum‐based drugs, such as platinum combined with pemetrexed for adenocarcinoma and platinum combined with paclitaxel for squamous cell carcinoma. Essential patient details, encompassing age, sex, smoking history, histology, T stage, N stage, M stage, and PD‐L1 expression level, were extracted from the patients' medical records. Regular follow‐up examinations occurred every 1–2 months to evaluate their condition. These follow‐ups included contrast‐enhanced chest CT, abdominal ultrasonography, contrast‐enhanced brain MRI, bone scan, or PET‐CT. Progression‐free survival (PFS) was defined as the duration from the commencement of immune‐chemotherapy to either the date of progression or the last follow‐up. Serum samples for nontargeted metabolomics testing were collected within 7 days before initiating ICIs in combination with chemotherapy.

### 2.3 Untargeted metabolomics methods

Serum samples, collected prior to combined treatment, underwent untargeted metabolomics analysis employing liquid chromatography‐mass spectrometry (LC–MS/MS). In this investigation, 55 serum samples were chosen, divided into two groups for metabolic studies within the same experimental conditions and utilizing identical experimental methods. Subsequent to sample extraction via methanol‐assisted protein precipitation, raw data were acquired and onboard data preprocessing was executed using Analyst TF 1.7.1 software (Sciex, Concord, ON, Canada) in IDA mode. To ensure precision, quality control samples (QC) were generated by blending sample extracts, with one QC sample typically integrated into every 10 test analysis samples during instrument analysis to oversee the repeatability of the analysis process.

### 2.4 Statistical analysis

Data analysis for untargeted metabolomics primarily encompasses principal component analysis (PCA), orthogonal partial least squares discriminant analysis (OPLS‐DA), fold change (FC), and differential metabolite functional annotation. PCA analysis was conducted using the inherent statistical function (prcomp) in R software to assess the experiment's stability, wherein QC samples exhibit enhanced data quality when more concentrated. OPLS‐DA was employed to establish a relationship model between metabolite expression and sample categories, facilitating sample category prediction. Variable Importance in Projection (VIP) >1, derived from the OPLS‐DA model's variable importance projection, concurrently meeting the *p*‐value <0.05 for univariate analysis, served as the criterion for preliminary differential metabolite screening in this study. FC values, determined through the *t*‐test, allowed the comparison of metabolite relative content between the two sample groups. The Kyoto Encyclopedia of Genes and Genomes (KEGG) database was utilized for a comprehensive annotation of all potential metabolic pathways of metabolites.

Chi‐square testing analyzed the association of M1c1 and M1c2 with patients' basic characteristics. Median PFS was computed using Kaplan–Meier, and group comparisons employed the log‐rank test. Cox regression analysis evaluated independent predictors and the impact of each factor on PFS. Statistical significance was defined as two‐sided *p* < 0.05, and the statistical analyses were conducted using SPSS 24.0 and GraphPad Prism 8.0 software.

## 3 RESULTS

### 3.1 Patient characteristics

A total of 55 patients meeting the inclusion and exclusion criteria participated in this study, with 33 (60.0%) categorized as M1c1 in the M stage and 22 (40.0%) classified as M1c2 in the M stage. The median PFS for all patients was 13.0 months, with a 95% confidence interval (CI) of 10.8–15.2. The baseline characteristics, including age, gender, smoking history, histology, T stage, N stage, and PD‐L1 expression, were well‐balanced between the M1c1 and M1c2 groups, as outlined in Table 1.

**TABLE 1:** Patients' Baseline Characteristics.

Characteristic	M1c1 (*n* = 33)	M1c2 (*n* = 22)	*p*	Total
Age (years), *n* (%)				
<65	7 (21.2)	10 (45.5)	0.057	17 (30.9)
≥65	26 (78.8)	12 (54.5)		38 (69.1)
Gender, *n* (%)				
Male	29 (87.9)	19 (86.4)	1.000	48 (87.3)
Female	4 (12.1)	3 (13.6)		7 (13.7)
Smoking history, *n* (%)				
Never	8 (24.2)	7 (31.8)	0.537	15 (27.3)
Current/former	25 (75.8)	15 (68.2)		40 (72.7)
Histology, *n* (%)				
Squamous	22 (66.7)	15 (68.2)	0.907	37 (67.3)
Adenocarcinoma	11 (33.3)	7 (31.8)		18 (32.7)
T stage, *n* (%)				
1–2	16 (48.5)	9 (40.9)	0.126	25 (45.5)
3–4	17 (51.5)	13 (59.1)		30 (54.5)
N stage, *n* (%)				
0–1	8 (24.2)	3 (13.6)	0.536	11 (20.0)
2–3	25 (75.8)	19 (86.4)		44 (80.0)
PD‐L1 expression, *n* (%)				
TPS <1%	8 (24.2)	5 (22.8)	0.763	13 (23.6)
1% ≤ TPS ≤ 49%	8 (24.2)	7 (31.8)		15 (27.3)
TPS ≥ 50%	9 (27.3)	7 (31.8)		16 (29.1)
Unknown	8 (24.3)	3 (13.6)		11 (20.0)

Abbreviations: PD‐L1, programmed cell death‐ligand 1; TPS, tumor proportion score.

### 3.2 Different prognoses in M1c1 and M1c2 patients

**FIGURE 1:**
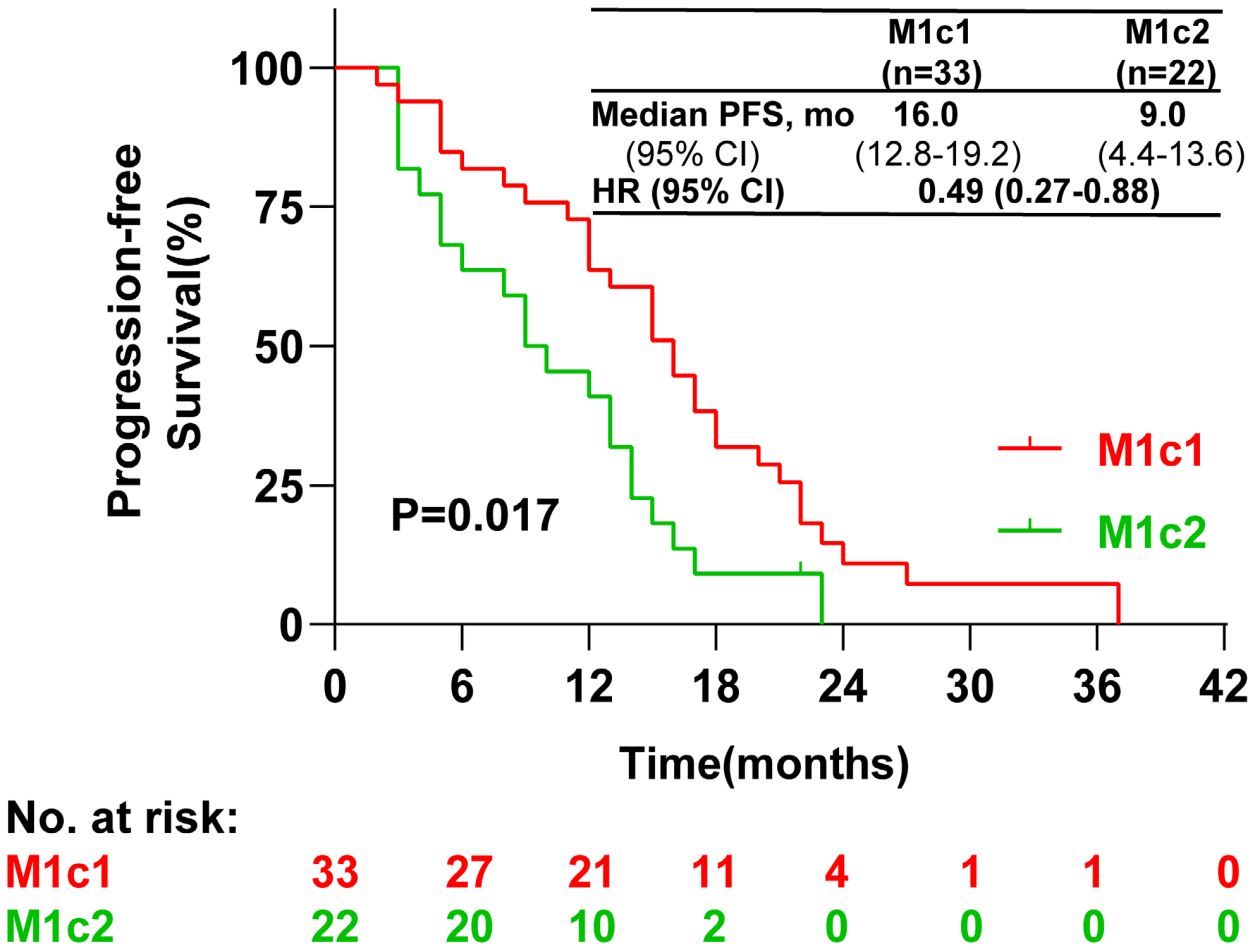
Kaplan–Meier Curves for PFS based on M1c1 and M1c2. PFS, progression‐free survival; mo, months; HR, hazard ratio.

**FIGURE 2:**
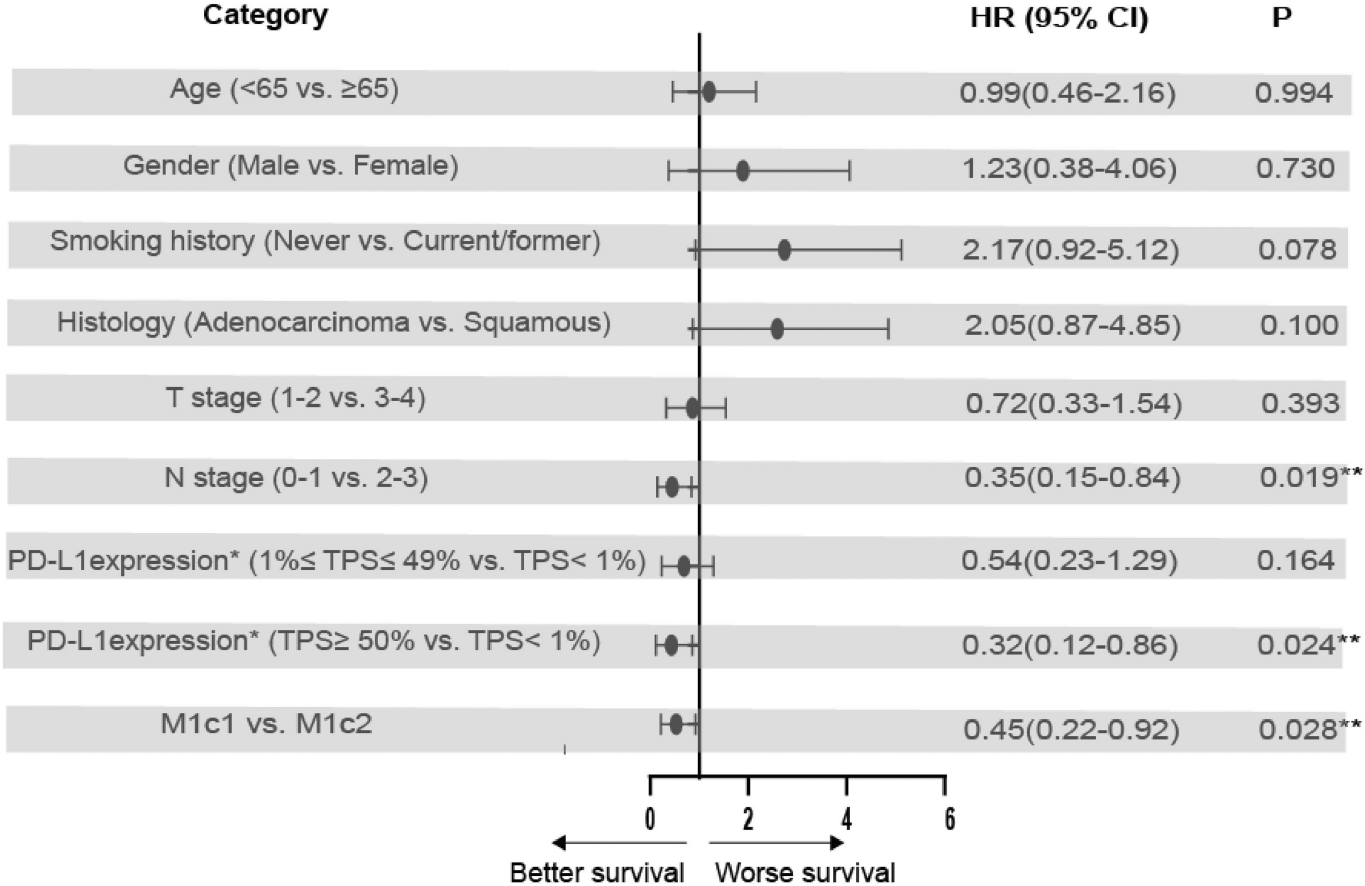
Forest Plot for Multivariate Cox Analysis of PFS in Patients. *Only for patients with available PD‐L1 expression data (patients with unknown PD‐L1 expression were excluded). PFS, progression‐free survival; HR, hazard ratio; PD‐L1, Programmed cell death‐ligand 1; TPS, tumor proportion score; **p < 0.05 indicates statistical significance.

As depicted in Figure 1, the median PFS was 16.0 months (95% CI: 12.8–19.2) for patients classified as M1c1 and 9.0 months (95% CI: 4.4–13.6) for those classified as M1c2, with a statistically significant difference (*p* = 0.017). Univariate analysis indicated no significant disparities in PFS across age, gender, smoking history, histology, T stage, N stage, and PD‐L1 expression groups (*p* > 0.05). However, PFS was notably prolonged in M1c1 patients compared to M1c2 (HR = 0.49, 95% CI: 0.27–0.88, *p* = 0.017), as detailed in Table 2. To explore independent prognostic factors, we incorporated age, sex, smoking history, histology, T stage, N stage, PD‐L1 expression, and M1c classification into Cox proportional hazards multivariate models. The multivariate analysis (Figure 2) revealed that age, gender, smoking history, histology, and T stage were not independent prognostic factors for PFS. Additionally, there was no survival difference between patients with PD‐L1 expression between 1 and 49% and those with PD‐L1 expression less than 1%. Notably, patients with N0‐1 and PD‐L1 expression ≥50% exhibited an extended PFS, and those with M1c1 receiving first‐line ICIs combined with chemotherapy experienced significantly improved prognosis compared to M1c2 patients (HR = 0.45, 95% CI: 0.22–0.92, *p* = 0.028).

**TABLE 2:** Univariate Analysis for PFS.

Variable	Univariate	
	HR (95% CI)	*p* value
Age (<65 vs. ≥65)	0.93 (0.51–1.67)	0.799
Gender (Male vs. Female)	1.26 (0.50–3.20)	0.623
Smoking history (Never vs. Current/former)	1.87 (0.96–3.62)	0.066
Histology (Adenocarcinoma vs. Squamous)	1.35 (0.74–2.45)	0.325
T stage (1–2 vs. 3–4)	0.85 (0.48–1.48)	0.555
N stage (0–1 vs. 2–3)	0.50 (0.23–1.09)	0.080
PD‐L1 expression^a^ (1% ≤ TPS ≤ 49% vs. TPS <1%)	0.52 (0.24–1.15)	0.107
PD‐L1 expression^a^ (TPS ≥ 50% vs. TPS <1%)	0.47 (0.21–1.03)	0.060
M1c1 vs. M1c2	0.49 (0.27–0.88)	0.017**

*Note*: **p < 0.05 indicates statistical significance.

Abbreviations: HR, hazard ratio; PFS, progression‐free survival; PD‐L1, programmed cell death‐ligand 1; TPS, tumor proportion score.

^a^ Only for patients with available PD‐L1 expression data (patients with unknown PD‐L1 expression were excluded).

### 3.3 Preliminary selection of differential metabolites

**FIGURE 3:**
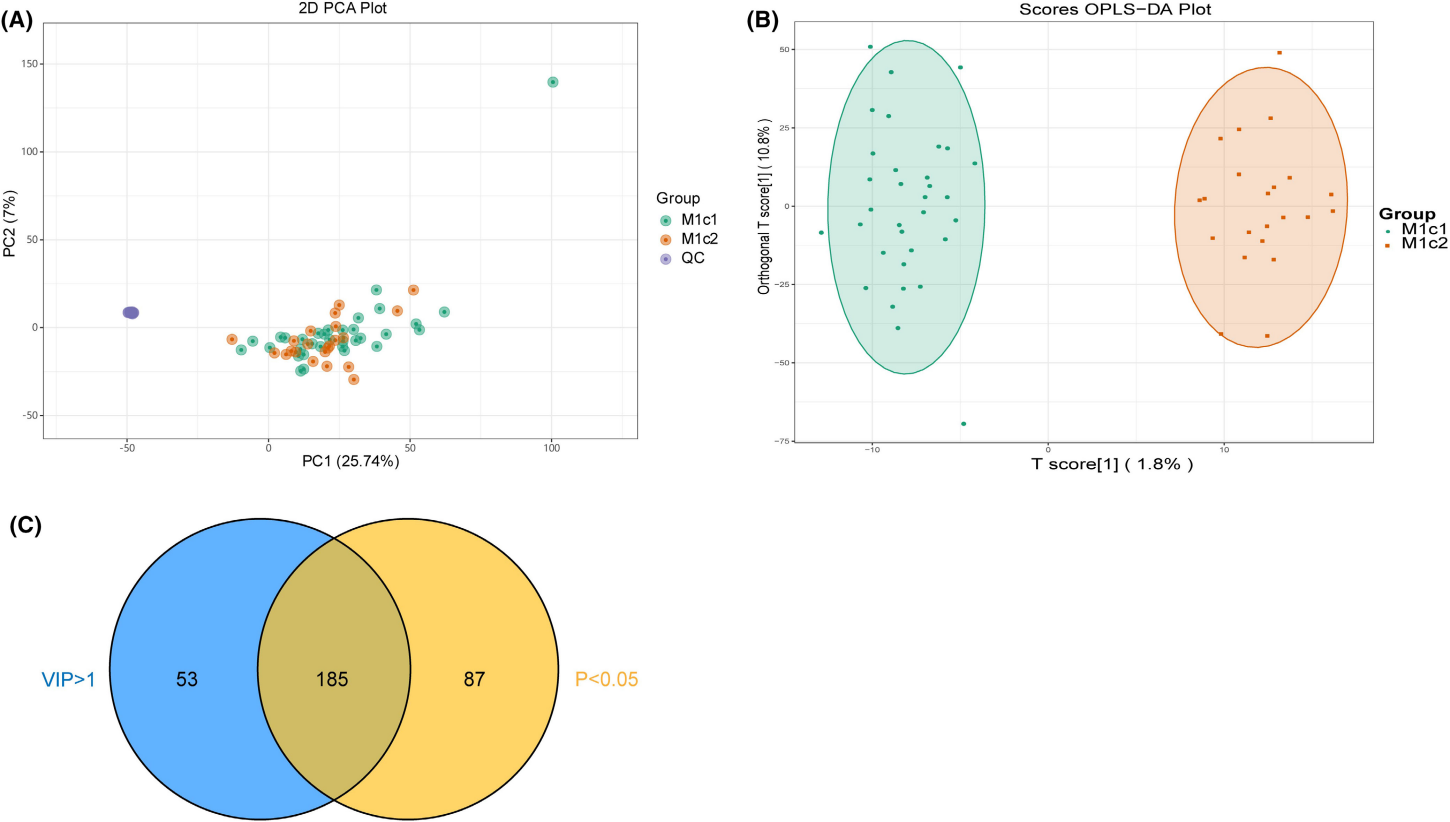
(A) PCA plots, (B) OPLS‐DA plots, (C) Venn plots for M1c1 and M1c2 groups. PCA, principal component analysis; OPLS‐DA, orthogonal least squares discriminant analysis.

**FIGURE 4:**
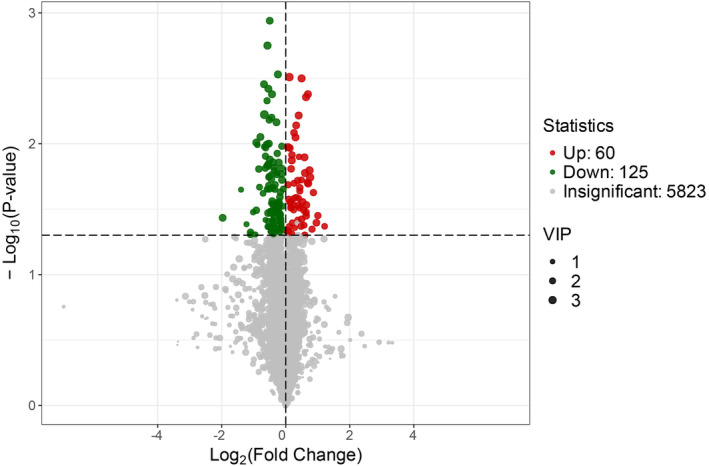
Volcano Plot of Differential Metabolites. Each point in the volcano plot represents a metabolite. The green point signifies a downregulated differential metabolite, the red point indicates an upregulated differential metabolite, and the gray point denotes a metabolite detected but not significantly different. The abscissa represents the logarithm of the fold difference in the relative content of a metabolite between the two groups of samples, with a greater absolute value indicating a larger difference in the relative content of the substance between the two groups of samples. The ordinate represents the level of significance of the difference, and the size of the dot represents the VIP value.

The PCA plot in Figure 3A reveals a close combination of QC samples, indicating the stability of the instrument analysis system and the reliability of the test data. The metabolic profile differences observed in the test accurately reflect the biological distinctions between the samples. However, the metabolomes of the M1c1 and M1c2 groups also exhibit a tendency to separate. The OPLS‐DA plot in Figure 3B accentuates the differences between the M1c1 and M1c2 groups, further enhancing group discrimination. Utilizing the OPLS‐DA model and VIP values, coupled with the criteria VIP >1 and *p* < 0.05, a preliminary selection of 185 differential metabolites was achieved. Among these, 125 were downregulated, and 60 were upregulated in the M1c2 group, as illustrated in Figure 4.

**FIGURE 5:**
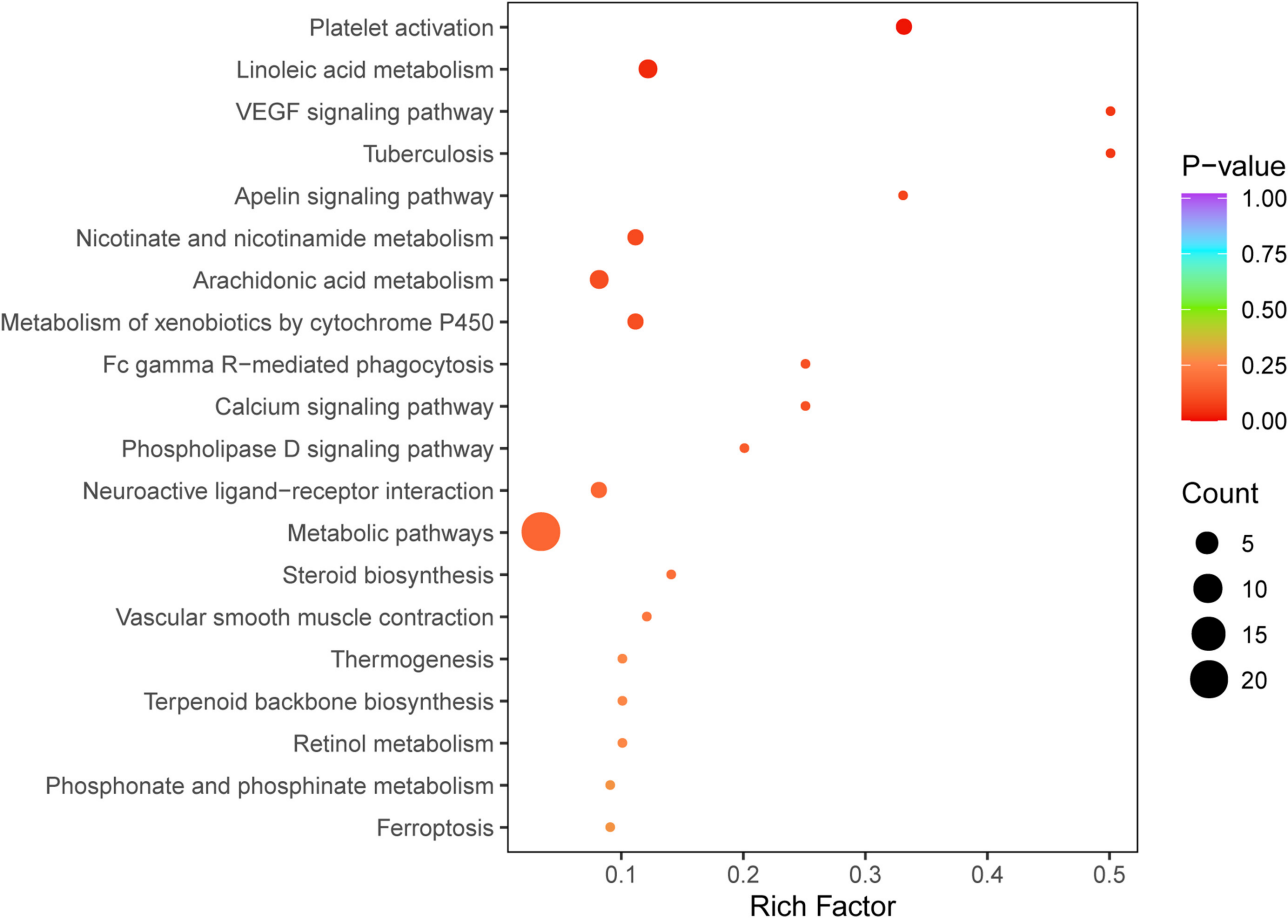
Differential Metabolite KEGG Enrichment Plot. KEGG, kyoto encyclopedia of genes and genomes. The abscissa represents the Rich Factor corresponding to each pathway, while the ordinate is the pathway name (sorted by *p*‐value from small to large). The color of the dots indicates the P‐value size, with red indicating more significant enrichment. The size of the dots reflects the number of differentially enriched metabolites.

Subsequently, differential metabolite results underwent KEGG pathway enrichment analysis, where the Rich Factor, representing the ratio of differential metabolites in a specific pathway to the total annotated metabolites, indicated the degree of enrichment. Larger values signified greater enrichment. The hypergeometric test *p*‐value, approaching 0, underscored the significance of enrichment. The top 20 pathways, ranked by *p*‐value from small to large, are presented in Figure 5. Notably, these differential metabolites were enriched in pathways linked to lung cancer metastasis and invasion, including platelet activation, linoleic acid metabolism, and the VEGF signaling pathway.

### 3.4 Search for clinically significant differential metabolites

**FIGURE 6:**
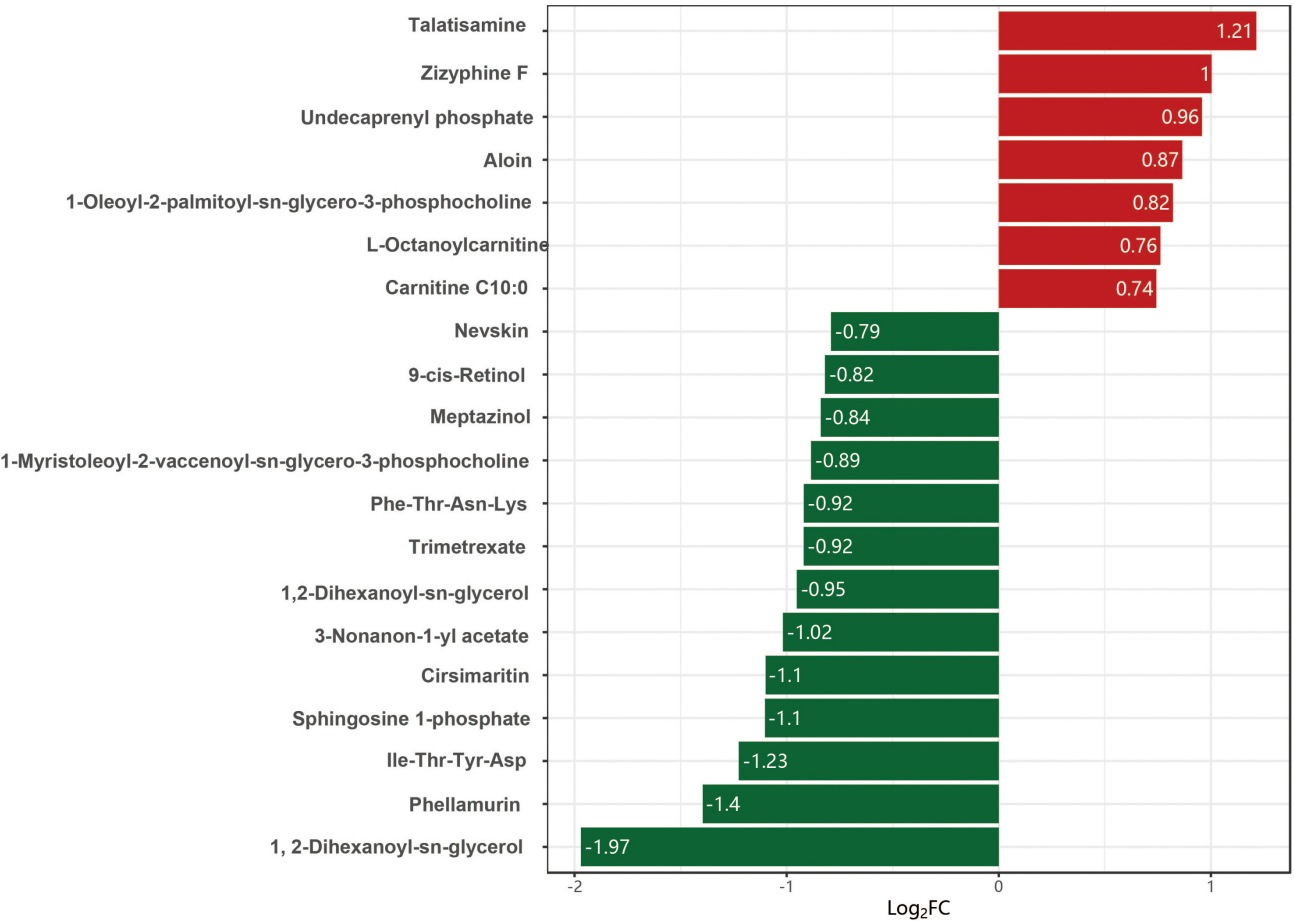
Fold Change (FC) Bar Chart (Top 20 Metabolites with FC Difference). FC, Fold change; log_2_FC, Fold difference values for differential metabolites taking the logarithm as base 2. Red represents upregulated metabolite content, and green represents downregulated metabolite content.

**FIGURE 7:**
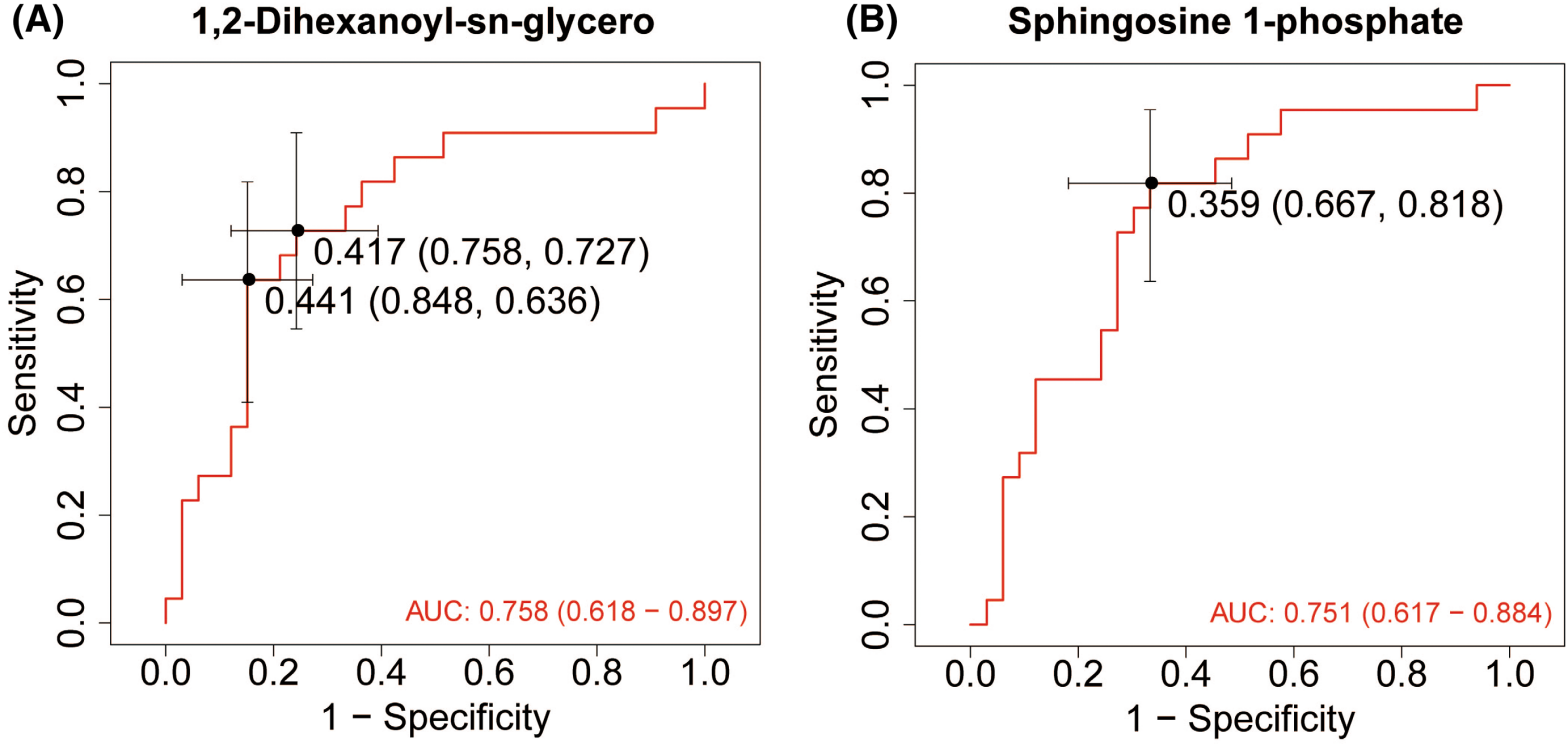
Receiver Operating Characteristic (ROC) Curves for Substances (A) 1,2‐Dihexanoyl‐sn‐glycerol and (B) Sphingosine 1‐phosphate. AUC, area under the curve.

**FIGURE 8:**
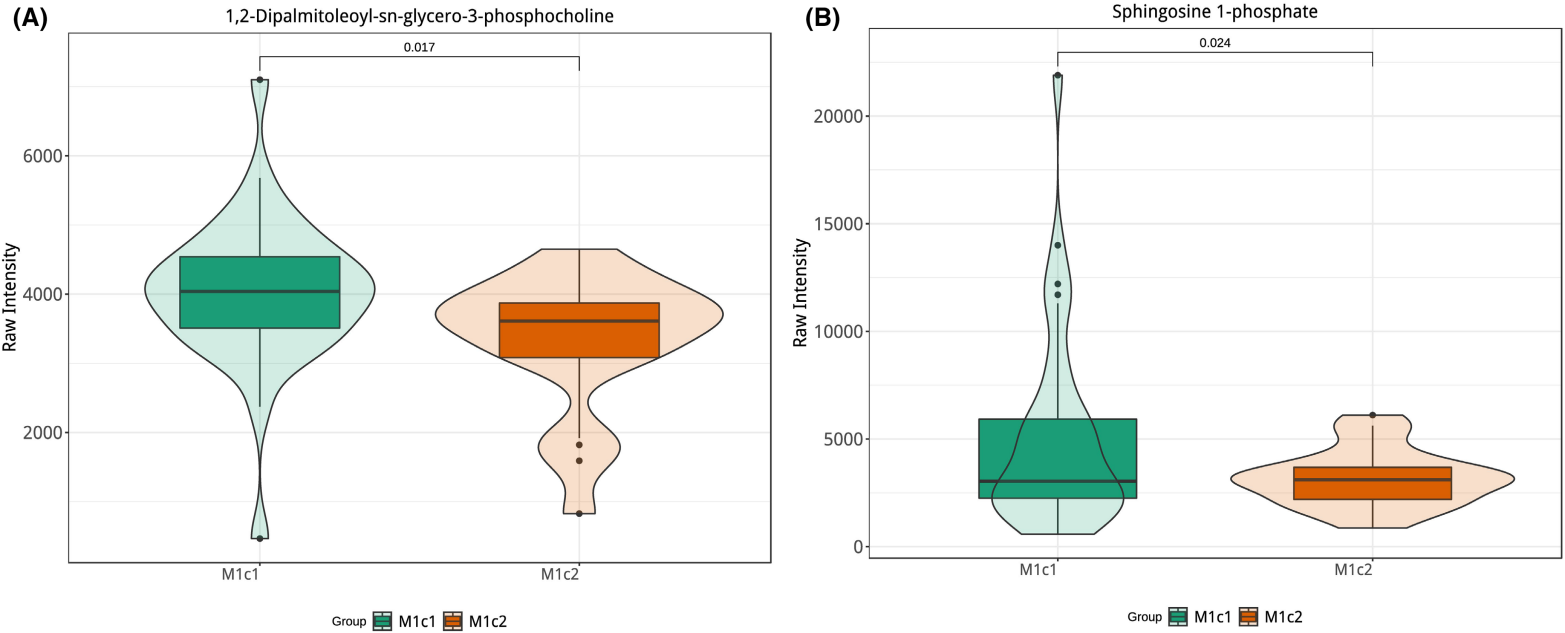
Violin Plots of Differential Substances (A) 1,2‐Dihexanoyl‐sn‐glycerol and (B) Sphingosine 1‐phosphate in M1c1 and M1c2 Groups.

Despite the initial screening yielding numerous metabolites (VIP >1 & *p* < 0.05), we implemented more stringent criteria (FC >2 & FC <0.5) to precisely identify differential metabolites. Eight metabolites met the criteria, with talatisamine and zizyphine F upregulated, and 3‐Nonanon‐1‐yl acetate, Cirsimaritin, Sphingosine 1‐phosphate, Ile‐Thr‐Tyr‐Asp, Phellamurin, and 1,2‐Dihexanoyl‐sn‐glycerol downregulated (Figure 6). Furthermore, we assessed the predictive performance of these metabolites using the area under the curve (AUC) values from receiver operating characteristic (ROC) curves (Table 3). Notably, in Figures 7, 1, and 2 Dihexanoyl‐sn‐glycerol and Sphingosine 1‐phosphate demonstrated superior prediction performance (AUC >0.7). In Figures 8, 1, and 2 Dihexanoyl‐sn‐glycerol, a glycerolipid, exhibited a significant decrease in the M1c2 group (*p* = 0.017), while Sphingosine 1‐phosphate, a sphingomyelin substance, also showed a significant decrease in the M1c2 group (*p* = 0.024).

**TABLE 3:** Area under the curve of potential metabolite biomarkers.

Compounds	AUC	95% CI
Talatisamine	0.638	0.485–0.791
Zizyphine F	0.685	0.542–0.827
3‐Nonanon‐1‐yl acetate	0.649	0.500–0.798
Cirsimaritin	0.672	0.527–0.817
Sphingosine 1‐phosphate	0.751	0.617–0.884
Ile‐Thr‐Tyr‐Asp	0.643	0.492–0.794
Phellamurin	0.660	0.508–0.812
1,2‐Dihexanoyl‐sn‐glycero	0.758	0.618–0.897

Abbreviation: AUC, area under the curve.

## 4 DISCUSSION

The latest iteration of the TNM staging criteria places renewed emphasis on the nuanced classification of patients with M1c into subcategories M1c1 and M1c2, rekindling vigorous debates within the academic community. TNM staging holds paramount significance in appraising malignancies, and the criteria for lung cancer have undergone continuous refinement in recent decades.^16^ Given the global prevalence of lung cancer, further subdivision of its staging may offer novel insights into treatment strategies and prognostic considerations. The current standard of care for patients with advanced driver gene‐negative NSCLC involves first‐line treatment with ICIs in combination with chemotherapy.^17^ It is crucial to investigate whether the prognosis differs between M1c1 and M1c2 patients receiving combination therapy, providing valuable real‐world data supplements for the application of the 9th edition of TNM staging and offering clinicians new references for treatment decisions in such cases.

Our study revealed a significant disparity in prognosis between M1c1 and M1c2 patients undergoing first‐line combined chemotherapy. Both univariate and multivariate analyses demonstrated a worse PFS in M1c2 patients compared to M1c1 patients. Furthermore, untargeted metabolomics analysis of pretreatment sera from these patient categories unveiled alterations in metabolic pathways, serving as a predictive tool for adverse outcomes. Notably, changes in lipid metabolism, specifically decreased glycerolipids and sphingomyelin, may be associated with a poorer prognosis in patients with tumor metastasis and invasion. Prior research has linked low glyceride content to diminished overall survival in colon cancer patients.^18^ Additionally, alterations in membrane composition, particularly a reduction in sphingomyelin content, have been observed in NK cells within the tumor microenvironment of advanced cancer patients. This alteration leads to a loss of NK cell processes, impairing their ability to recognize and eliminate tumor cells.^19^

Confirmation of the survival curves for M1c1 and M1c2 patients, distinctly separated, constitutes a key observation in our study. The primary focus was on discerning whether the prognosis of M1c1 and M1c2 patients receiving first‐line PD‐1/PD‐L1 inhibitors combined with chemotherapy differed, a facet not previously demonstrated in existing research. Upon plotting survival curves, a significant divergence was evident, with M1c1 patients exhibiting a markedly longer median PFS compared to M1c2 patients. Recognizing the potential influence of factors such as age, sex, T stage, N stage, and PD‐L1 expression on patient prognosis, both multivariate and univariate analyses were conducted for these variables alongside M1c classification. Univariate analysis revealed a significantly superior PFS in M1c1 patients, while other factors did not exhibit a significant association. Multivariate analysis identified N stage, PD‐L1 expression, and M1c classification as independent predictors, underscoring the meaningful contribution of further refining the classification of M1c. N stage, a well‐established prognostic factor in lung cancer, and PD‐L1 expression, a reliable predictor of immunotherapy outcomes, emerged as critical factors impacting the prognosis of lung cancer patients undergoing immune combination therapy.

Metabolomics has progressively emerged as a cutting‐edge technology extensively employed in the identification of disease biomarkers in recent years.^20^, ^21^ In comparison to preceding genomics and proteomics approaches, metabolomics offers simpler technical methodologies, facilitating more convenient application in clinical practice. Notably, the detection of species changes is straightforward, the number of changes is limited, and verification and analysis are easily conducted. Crucially, alterations in metabolic levels can provide real‐time insights into the physiological and pathological state of the body.^22^ While PD‐L1 expression has been acknowledged as a predictive biomarker for immunotherapy, its limitation lies in the lack of dynamic monitoring of bodily changes. In our study, we utilized untargeted metabolomics testing of serum samples, enabling the dynamic reflection of changes in the physiological and pathological status of the body. This approach also allowed the exploration of a broad spectrum of potential biomarkers.^23^ Despite the established association of untargeted metabolomics with lung cancer, its application to investigating metabolic differences between M1c1 and M1c2 patients, particularly in the context of immune combination therapy efficacy, represents a novel approach. By collecting serum samples before treatment and conducting untargeted metabolomics testing, our study identified multiple changes in metabolic pathways associated with cancer metastasis in both patient subgroups. Notably, we uncovered that alterations in lipid‐associated metabolites may contribute to distinct prognoses in these patients. This innovative aspect of our study contributes valuable insights to the intersection of metabolomics, lung cancer, and immune combination therapy.

Research has clarified the crucial role of lipid metabolism in controlling various aspects of cancer biology, including cell growth, survival, proliferation, migration, invasion, and metastasis. A study on melanoma cells highlighted lipid metabolism‐related pathways as predominant among the top five differential pathways, emphasizing oleic acid lipids as major contributors.^24^ Another examination of early NSCLC revealed widespread abnormalities in lipid metabolism across diverse cell types. Disturbed pathways included glycerol lipid metabolism, glycerophospholipid metabolism, fatty acid biosynthesis, and unsaturated fatty acid biosynthesis.^25^ Abnormal lipid metabolism not only affects oncogenic signaling pathways within cancer cells but also influences neighboring normal cell populations through secretory components, including lipids. In our study, the observed reduction in glycerolipid and sphingolipid metabolites in the serum of M1c2 patients with a poor prognosis suggests a potential link to mechanisms through which enhanced tumor metastasis may be associated with increased fat consumption. Tumor cells, especially when metastasizing or developing resistance to treatment, intensify their lipid absorption, lipid oxidation, and lipid synthesis mechanisms. This enhancement can be further fueled by excessive fat consumption.^26^ However, it is essential to acknowledge the complexity of lipid metabolism. Changes in lipids within the primary tumor microenvironment and the premetastatic microenvironment play crucial roles in facilitating the escape and dissemination of cancer cells, as well as evasion of immune surveillance. The intricate interplay of lipid metabolism in cancer progression underscores its multifaceted impact on tumor behavior and treatment response. Additionally, altered lipid metabolism may reflect the development of cachexia. Cachexia is one of the major complications and causes of death in cancer patients, resulting in severely weakened tumor treatment outcomes. Although the etiology of cachexia is very complex, it has been shown that the immune system and carbohydrate, protein, fat and energy metabolism interactions are one of the processes of cachexia formation.^27^ During cachexia, lipolysis increases while lipid uptake and lipogenesis are suppressed and lipid droplets from adipose tissue are depleted, leading to an imbalance in lipid metabolism.^28^

However, this study has limitations. Firstly, the sample size is small, and a larger cohort is required to validate our conjecture. And our data focused on only M1c disease, which might be biased. We hope, in the future, we could include data from M1a, M1b disease for comparison. Additionally, retrospective studies have constraints, and the follow‐up time is insufficient to observe the overall survival of patients. Therefore, longer follow‐up and prospective studies may enhance the accuracy of the results. Although untargeted metabolome assays can identify numerous differential metabolites, subsequent validation experiments are necessary.

Our study identified differences in the prognosis of NSCLC patients with M1c1 and M1c2 receiving first‐line immunotherapy plus chemotherapy. The prognosis of M1c1 patients was significantly better than that of M1c2. Untargeted metabolomics of serum revealed a distinct separation of metabolic profiles and some predictability of outcomes in these two patient categories. Importantly, we observed altered lipid metabolism, with reductions in diacylglycerol 1,2‐Dihexanoyl‐sn‐glycerol and sphingomyelin Sphingosine 1‐phosphate associated with adverse outcomes. Metabolomics‐derived abnormalities in lipid metabolism may contribute to a better understanding of the causes of cancer metastasis and offer therapeutic potential for cancer treatment.

## AUTHOR CONTRIBUTIONS

**Liang Zheng:** Writing – original draft (equal). **Fang Hu:** Conceptualization (equal). **Wei Nie:** Data curation (equal). **Jun Lu:** Data curation (equal). **Bo Zhang:** Visualization (equal). **Jianlin Xu:** Visualization (equal). **Shuyuan Wang:** Methodology (equal). **Ying Li:** Methodology (equal). **Xiaoxuan Zheng:** Methodology (equal). **Wei Zhang:** Resources (equal). **Yinchen Shen:** Resources (equal). **Runbo Zhong:** Resources (equal). **Tianqing chu:** Project administration (equal). **Baohui Han:** Project administration (equal). **Hua Zhong:** Project administration (equal). **Xueyan Zhang:** Writing – review and editing (equal).

## FUNDING INFORMATION

This study was financially supported in part by Shanghai Innovative Medical Device Application Demonstration Project 2023, Grant Number: 23SHS02600; National Natural Science Foundation of China, Grant Number: 82373425; the Medical Innovation Research Special Project of the Science and Technology Commission of Shanghai Municipality: 23Y11904200; the Fundamental Research Funds for the Central Universities, Grant Number: YG2023QNB23.

## CONFLICT OF INTEREST STATEMENT

The authors declare that they have no known competing financial interests or personal relationships that could have appeared to influence the work reported in this paper.

## Data Availability

Data available on request from the authors.
